# How does water stress affect the bioaccumulation of galanthamine and lycorine, growth performance, phenolic content and defense enzyme activities in summer snowflake (*Leucojum aestivum* L.)?

**DOI:** 10.1007/s12298-024-01451-8

**Published:** 2024-04-29

**Authors:** Yavuz Baba, Ayca Cimen, Arzu Birinci Yildirim, Arzu Ucar Turker

**Affiliations:** 1 Department of Biology, Faculty of Science and Art, Bolu Abant Izzet Baysal University, 14030 Bolu, Türkiye; 2 Department of Field Crops, Faculty of Agricultural and Environmental Science, Bolu Abant Izzet Baysal University, 14030 Bolu, Türkiye

**Keywords:** Galanthamine, *Leucojum aestivum*, Lycorine, PEG, Water stress, Waterlogging

## Abstract

*Leucojum aestivum* L. is an Amaryllidaceae bulbous plant with two alkaloids that have remarkable medicinal potential: galanthamine and lycorine. Although the presence of galanthamine in *L. aestivum* has commercial value for the pharmaceutical industry and the effect of water stress (WS) applications on secondary metabolite enhancement is well established in a variety of plants, no studies have been carried out to reveal the effectiveness of WS on this beneficial medicinal plant. Objective of the study was to investigate the effects of eight different WS treatments [Control, waterlogging (WL) condition, and drought stress conditions (water deficiency generated by water deficit irrigation-WDI 25%, 50%, and 75%- and polyethylene glycol-PEG 6000 15%, 30%, and 45%-)] on growth parameters, alkaloid levels (galanthamine and lycorine), non-enzymatic antioxidant activities (total phenol-flavonoid content and free radical scavenging activity), and enzymatic antioxidant activities [superoxide dismutase (SOD) and catalase (CAT)] of *L. aestivum* in a pot experiment. Based on the findings, maximum increases in growth parameters were obtained with PEG-induced WS treatments. Moderate water deficiency (50% WDI) produced the highest levels of galanthamine and lycorine, total phenol-flavonoid content, and antioxidant capacity, along with moderately elevated CAT activity in the bulbs. All WS treatments resulted in increased CAT activity in the bulbs. It was observed that bulbs had higher SOD and CAT activities under WL conditions had lower fresh weights and were close to control in terms of alkaloid levels, total phenol-flavonoid content, and free radical scavenging activity. When all of the outcomes were taken into account, it can be concluded that moderate water-deficit stress (50% WDI) was regarded as the most effective treatment for increasing the pharmaceutical value of *L. aestivum*.

**Graphical abstract:**

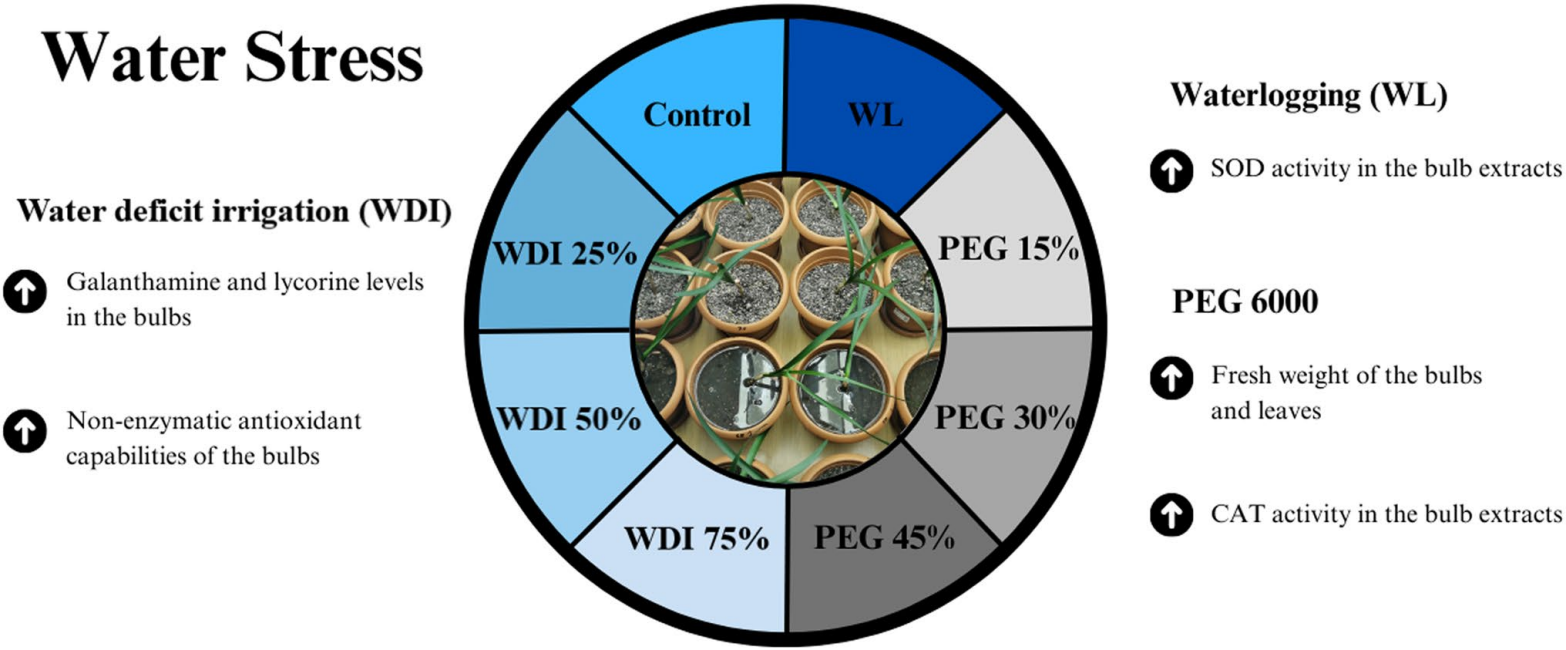

## Introduction

The summer snowflake*, Leucojum aestivum* L., is a bulbous Amaryllidaceae family member. It is a protected medicinal and ornamental plant native to South Africa, the Mediterranean, parts of Eastern Europe, and Western Asia (Ates et al. 2021; Demir et al. 2022). It grows from sea level to high elevations in semi-shaded and humid environments such as swamps, marshes, and floodplains (Demir et al. 2022). Many natural *L. aestivum* habitats have deteriorated or are endangered during the last three decades due to the increasing demand from pharmaceutical businesses (Ivanov et al. 2019). *L. aestivum* produces alkaloids with noteworthy pharmacological characteristics. Galantamine, an isoquinoline alkaloid acetylcholinesterase inhibitor, is a significant medication used globally for the symptomatic treatment of Alzheimer's disease-related senile dementia (Diop et al. 2007), poliomyelitis, and other neurological illnesses (Pavlov et al. 2007). Lycorine had potent antiviral activity against poliovirus, measles, and Herpes simplex type 1 virus, in addition to high antiretroviral, antimalarial, antimitotic, and cytotoxic properties (Saliba et al. 2015). It is a powerful, non-nucleoside, direct-acting antiviral against developing coronavirus infections (Jin et al. 2021).

There are two circumstances that can cause water stress (WS): either an excess of water or a deficiency of water. Drought stress, or water-deficit stress, is the more typical type of WS (Mahajan and Tuteja 2005)**.** Plants are stationary organisms unable to circumvent environmental constraints (Aroca et al. 2012). The plant environment has the greatest influence on herbage quality by affecting leaf/stem ratios, but it also causes other morphological changes and modifications in the chemical composition of the plant parts (Buxton and Fales 1994). By modifying their cellular metabolism and activating multiple defensive systems, plants may respond to and adapt to WS (Jiang 2002). In general, drought stress occurs when soil moisture is depleted, and atmospheric conditions promote a constant loss of water through transpiration or evaporation (Jaleel et al. 2009). Polyethylene glycol (PEG-6000) molecules are inert, non-ionic, and almost impermeable chains; PEG-6000 molecules are small enough to affect osmotic potential but big enough to prevent absorption by plants (Van den Berg and Zeng 2006). PEG-6000 solutions have been utilized efficiently to simulate the drought stress with fewer metabolic interferences than those associated with the use of low molecular weight osmolytes that can be absorbed by the plant (Muscolo et al. 2014). Plants engage their drought response mechanisms, including morphological and structural changes, the activation of drought-resistant genes, the manufacture of hormones, and osmotic-regulating chemicals to reduce drought stress (Yang et al. 2021).

Flooding stress typically encompasses two types of situations: submergence stress, when the plant is entirely submerged, and waterlogging stress, where the plant's leaf and stem are partially submerged. In many places and circumstances, waterlogging, a significant environmental stress, severely limits crop growth and output (Mahajan and Tuteja 2005). Waterlogging stress typically results from external environmental variables such as a lot of rain, too much irrigation, storms, and rivers overflowing. If there is freestanding water on the soil surface or if the available water fraction of the top layer is at least 20% greater than the field capacity, the soil is deemed to be waterlogged (Ahsan et al. 2007; Tewari and Mishra 2018; Zhang et al. 2021). Flooding is an example of an overabundance of water, which largely affects the roots' ability to receive oxygen. Critical root processes, such as limited food uptake and respiration, become dysfunctional as a result of low oxygen levels (Mahajan and Tuteja 2005).

At the cellular level, drought stress frequently results in a buildup of reactive oxygen species (ROS). Excessive ROS generation can result in oxidative stress on the photosynthetic apparatus and severely impede normal cell activity. The increased number of ROS can be considered a danger to the cell, but they can also participate in the stress signal transduction pathway as secondary messengers. The capacity of plants to scavenge ROS and reduce their detrimental effects may correlate with their drought tolerance (Deeba et al. 2012). Plants have their own antioxidant system. This system is comprised of low molecular weight antioxidants, including phenolic compounds, ascorbate, glutathione, α-tocopherol, and carotenoids, as well as many enzymes, including superoxide dismutase (SOD), catalase (CAT), peroxidase (POD), ascorbate peroxidase (APX), and glutathione reductase (GR) (Saglam et al. 2011).

Under conditions of extreme water scarcity, the restriction of water passage from the xylem to the surrounding elongating cells can limit cell elongation in higher plants. Under drought conditions, impaired mitosis, cell elongation, and expansion limit plant height, leaf area, and crop growth (Farooq et al. 2009). To maintain growth and productivity under drought-stress circumstances, plants employ a variety of mechanisms, including increased synthesis of secondary metabolites and phytohormones, ROS signaling, plant hydraulic status, and osmotic adjustment. Plants produce primary metabolites for vital processes such as growth and development, as well as secondary metabolites for specialized purposes. Under unfavorable environmental circumstances, plants manufacture a substantial quantity of secondary metabolites necessary for survival (Yang et al. 2018; Yadav et al. 2021). While the presence of galanthamine in *L. aestivum* has commercial value for the pharmaceutical sector and the effect of WS applications on secondary metabolite enhancement is well established in diverse plants, to our knowledge, no previous studies have been performed to reveal the efficacy of WS on this valuable plant. Based on previous findings on the positive effects of WS on different plants, it was aimed to investigate the effects of eight different WS treatments on the levels of alkaloids (galantamine and lycorine), growth and development, and enzymatic (SOD and CAT) and non-enzymatic (DPPH free radical scavenging potency and total phenol-flavonoid content) antioxidant activities in the bulbs and leaves of *L. aestivum*.

## Material and methods

### WS treatments in pot culture

**Fig. 1:**
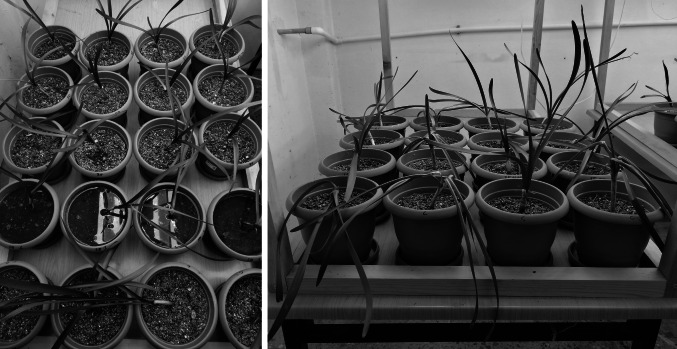
*L. aestivum* in pots with WS treatments. All eight WS treatments were completely randomized in pots of four (a total of 32). The experiment was repeated twice

*L. aestivum* bulbs were gathered from natural habitat (Bolu-Gölcük) in March at the vegetative stage when they had reached about 5 cm in length (Mill 1984). Nearly the same size of the *L. aestivum* bulbs was chosen randomly and planted into the pots (18.5 cm × 15.5 cm) containing the soil mixture, including 4:1:1 (v:v) ratios of peat (Terradena®, 65% peat, and 35% soil), sand, and vermiculite (Agrekal®), respectively (Fig. 1). The maximum pot water holding capacity (PWHC) was estimated using the method given by Gutiérrez-Miceli et al. (2007). After watering all pots in the experiment with the amount determined by PWHC (500 ml), irrigation water (IW) and watering frequency were determined by checking every day using a soil moisture meter (Extech Instruments®, MO750). The amount of IW was 150 ml, and the irrigation period was determined to be once a week. The electrical conductivity (EC) and pH (inoLab pH 7110, WTW, Weilheim, Germany) of the medium mix were measured as 1.62 µc/cm and 7.3, respectively. Bulbs were planted into pots for a 2-week acclimation phase. After acclimation phase, pots were set up for the establishment of experimental groups. Eight different WS treatments were established (Table 1; Fig. 1). The experimental design was completely randomized in pots of four (32 in total) for all WS treatments. The experiments were carried out twice.

**Table 1:** Establishment of eight different WS treatments

WS treatments	Irrigation amount (ml/pot/week)	Application
WS1 (C)	150	Only specified IW was supplied to pots
WS2 (WL)	150	Pots with no holes at the bottom were used and 300 ml water was supplied to obtain water level 4–5 cm above the soil surface to imitate the flooding in the third week
WS3 (WDI 25%)	112.5	The amount of IW was reduced by 25% in the third week (watering with 75% of IW)
WS4 (WDI 50%)	75	The amount of IW was reduced by 50% in the third week (watering with 50% of IW)
WS5 (WDI 75%)	37.5	The amount of IW was reduced by 75% in the third week (watering with 25% of IW)
WS6 (PEG 6000 15%)*	150	Begin watering with specified IW containing 3% PEG 6000 in the third week
WS7 (PEG 6000 30%)*	150	Begin watering with specified IW containing 6% PEG 6000 in the third week
WS8 (PEG 6000 45%)*	150	Begin watering with specified IW containing 9% PEG 6000 in the third week

At the beginning of the experiment, after all pots received the amount of water (500 ml) specified by PWHC, pots were watered with determined IW (150 ml) for the second week (a 2-week acclimation period). All treatments were carried out for five weeks following the acclimatization phase

*At the end of 5 weeks, cumulatively, 3 different treatment groups containing 15%, 30%, and 45% PEG were established. Thus, the effect of 3 different osmotic potentials originating from PEG (− 0.30, − 1.04, and − 2.22 MPa, respectively) was investigated. The osmotic potential of PEG 6000 was calculated according toMichel and Kaufmann (1973)

Different WS applications were conducted in all experimental groups for 5 weeks and were harvested after 7 weeks in total with acclimatization. The pot experiment was carried out in a plant room at 22 ± 1 °C with a 16/8 h (light/dark) photoperiod (cool white, fluorescent lights, 22–28 µmol m 2 s^−1^) and 60% relative humidity.

At the completion of the WS treatments, bulbs and leaves were gathered individually after 7 weeks. The length, width, and weight of each bulb and leaf were measured separately. Until extraction and biological activity testing, all plant samples were lyophilized at − 65 °C (Christ®) and kept at − 20 °C.

### Methanolic extracts preparation

Plant materials were prepared as methanol (MeOH) extracts in a water bath at 40 °C. The extractions were filtered after 24 h, and then methanol was vacuum evaporated (Buchi® rotary evaporator) at 45 °C. To get the final concentration, the dried extract was dissolved once more in MeOH.

### Alkaloid content determination through HPLC

Utilizing an HPLC system (VWR-Hitachi LaChrom Elite®) equipped with a Hitachi L-2455 diode array detector (DAD), a Hitachi L-2130 pump, and a Hitachi L-2200 autosampler, the quantitative analysis of methanol extracts was identified. Two alkaloid standards [galanthamine hydrobromide (TCI America®) and lycorine (Sigma®)] were utilized as references (in 0.1% TFA), and their calibration curves (6.25, 12.25, 25, 50, 100, and 200 mg/L) were used to calculate the levels of these compounds in the plant extracts. The HPLC method was carried out in accordance with the guidelines provided by Arslan et al. (2020), and an isocratic elution was employed for the analysis.

### Investigations of non-enzymatic antioxidant activity

#### Total phenolic content (TPC) quantification

According to Turker et al. (2021), the total phenolic content (TPC) of *L. aestivum* extracts was measured utilizing a modified Folin-Ciocalteu assay. The TPC of the extracts was calculated using a calibration curve, with Gallic acid (Sigma®) serving as the phenol standard. The absorbance of each sample was measured at 765 nm against a blank using a UV–vis spectrophotometer (Hitachi U-1900®). The extracts' TPC was quantified as equivalent to mg of gallic acid (GAE) per 1 g of dried extract.

#### Total flavonoid content (TFC) quantification

Using Turker et al. (2021) modified aluminum colorimetric test, the total flavonoid content (TFC) of *L. aestivum* extracts was measured. The flavonoid standard used was quercetin (Sigma®), and a calibration curve was constructed to determine the TFC of the extracts. The absorbance of each sample was measured at 415 nm against a blank using a UV–vis spectrophotometer (Hitachi U-1900®). The extracts' TFC was reported in milligrams of quercetin equivalent (mg QE) per one gram of dried extract.

#### Free radical scavenging activity

The antioxidant capacity of *L. aestivum* extracts was identified spectrophotometrically using the 2,2-diphenyl-1-picrylhydrazil (DPPH, Sigma-Aldrich Chemie®, Steinheim, Germany) radical assay, using a modified version of the Blois (1958) method as described by Turker et al. (2021). The DPPH method, a 0.13 mM DPPH solution, a plant sample, and quercetin (as an antioxidant standard) were all dissolved in methanol. 100 ml of plant sample, quercetin at varying doses, and methanol were combined with 1400 ml of DPPH (as a control). After 30 min in the dark, the absorbance of the samples was measured at 517 nm using a UV–vis spectrophotometer (Hitachi U-1900®) against a blank (methanol).

### Enzymatic antioxidant activities

#### Protein identification and extraction of enzymes

For measuring the SOD and CAT enzyme activity, enzymes and proteins were obtained from the bulbs and leaves of *L. aestivum*. For the enzyme extraction technique, fresh plant samples were completely crushed into a powder in an ice bath using liquid nitrogen. 0.1 g of each powder was then separated and homogenized in 4 ml of 50 mM phosphate buffer (pH 7), which contained 2 mM Na-EDTA and 1% (w/v) polyvinyl polypyrrolidone (PVP). The homogenate was centrifuged for 15 min at 12,000 rpm and 4 °C, and the supernatant was evaluated for SOD and CAT enzyme activity. The Lowry method (Lowry 1951) has been used to identify the protein amount of plant bulbs and leaves. Bovine serum albumin (BSA) was utilized as a protein standard.

#### Activity of the superoxide dismutase (SOD) enzyme

Based on the work of van Rossum et al. (1997), a modified technique for detecting SOD activity has been developed. In test tubes, 1.425 ml of a reaction mixture containing 0.3 mM xanthine, 0.6 mM EDTA, 150 mM nitro blue tetrazolium (NBT), 400 mM sodium carbonate (Na_2_CO_3_), and 1 g/L BSA were added. After that, 0.025 ml of xanthine oxidase solution was added to each tube. After 20 min of incubation at 25 °C, the reaction was stopped by adding 0.05 ml of 0.8 mM copper chloride. Using a UV–vis Spectrophotometer (Hitachi U-1900®), the absorbances of plant samples were measured relative to distilled water at 560 nm. One unit of SOD is equal to the amount of protein that produces a 50% reduction in NBT in a reaction, and activity is demonstrated as one unit per mg of protein.

#### Activity of the catalase (CAT) enzyme

The CAT activity was measured using the method published by Lartillot et al. (1988) by detecting the reduction in absorbance at 240 nm induced by the catalase enzyme's breakdown of H_2_O_2_. A combination of 50 mM phosphate buffer and 10 mM H_2_O_2_ has been added to the test tubes in order to measure CAT activity. To initiate the reaction, 20 ml of enzyme extract have been added to the mixture, which has been incubated at 25 °C for 2 min. Lastly, the reaction was stopped by adding 0.5 ml of a 1 M HCl solution. In order to assess the CAT activity in each sample, the consumption of H_2_O_2_ at 240 nm for two minutes was utilized. The activity was computed using the H_2_O_2_ extinction value of 0.0392 mM/cm and represented as mmol H_2_O_2_/mg protein.

### Data analysis

The investigations have been designed using a completely randomized design. For data analysis, Analysis of variance (ANOVA) and Duncan's Multiple Range Tests using SPSS version 26 (IBM Corp, NY, USA) have been performed. All results in the tables are demonstrated as a mean number ± standard error (SE). Means with the same letter within columns are not significantly different at *P* > 0.05. Pearson correlation analysis was utilized to prove the relationship.

## Results and discussion

### Growth parameters and water content (%)

WS developed by WDI and PEG was found to significantly enhance the widths of the bulbs and leaves. Additionally, it was determined that the fresh weights of the bulbs and leaves were crucially increased only by the WS produced by PEG (Table 2). The treatments of 45% PEG and 15% PEG showed the largest bulb diameter compared to the control, with 24.20% and 16.82% rises, respectively. WDI treatment of 75% also elevated the leaf widths by 14.17%. Bulb and leaf lengths were reduced with WS applications compared to the control. While 30% PEG treatment resulted in a significant decrease in bulb lengths, 50% WDI treatment led to the most noticeable decrease in leaf lengths. Fresh weights showed a substantial rise with 45% PEG in the bulbs and 15% PEG application in the leaves compared to the control (36.57% and 5.00% increases, respectively) (Table 2).

**Table 2:** Effect of WS treatments on width, length, and fresh weights of individual *L. aestivum* bulbs and leaves

		*L. aestivum*					
WS treatments		Widths (mm)		Lengths (mm)		Fresh weights (g)	
		Bulb	Leaf	Bulb	Leaf	Bulb	Leaf
C		20.33 ± 0.49^ cd^	11.50 ± 0.15^ cd^	37.75 ± 1.18^a^	438.20 ± 13.15^a^	6.48 ± 0.17^bc^	8.23 ± 0.69^ab^
WL		21.50 ± 1.19^ cd^	10.08 ± 0.08^f^	35.67 ± 0.88^ab^	398.00 ± 7.06^bc^	3.78 ± 0.14^e^	6.03 ± 0.19^ cd^
WDI	25%	20.00 ± 1.08^d^	11.09 ± 0.31^de^	36.33 ± 2.03^a^	395.80 ± 4.20^bc^	4.58 ± 0.37^d^	7.52 ± 0.63^ab^
	50%	22.50 ± 0.87^bc^	10.60 ± 0.22^ef^	33.50 ± 0.50^b^	357.50 ± 8.81^d^	5.87 ± 0.26^c^	4.17 ± 0.07^e^
	75%	20.40 ± 0.51^ cd^	13.13 ± 0.35^a^	29.25 ± 0.48^c^	367.20 ± 21.37^ cd^	5.04 ± 0.18^d^	5.31 ± 0.06^de^
PEG 6000	15%	23.75 ± 0.48^ab^	12.50 ± 0.22^b^	30.50 ± 0.50^c^	405.75 ± 5.31^ab^	6.73 ± 0.34^b^	8.60 ± 0.36^a^
	30%	21.00 ± 0.63^ cd^	11.00 ± 0.19^de^	28.33 ± 0.33^c^	402.25 ± 6.30^bc^	6.11 ± 0.22^bc^	6.80 ± 0.31^bc^
	45%	25.25 ± 0.25^a^	12.07 ± 0.16^bc^	33.33 ± 0.33^b^	412.60 ± 8.49^ab^	8.85 ± 0.33^a^	8.27 ± 0.84^ab^

Data are means ± standard error of three replicates (n = 3). Means with the different letters within columns show significant difference according to Duncan test (*p* < 0.05)

No improvement in growth parameters was observed with WS generated by WL. Although the bulb width of the WL group was the same as the control, all other parameters declined in comparison to the control with the WL application. With WL treatment, the fresh weight of the bulbs and leaf widths decreased the most (Table 2). The reason might be that plants growing in soggy soil were subjected to stressful conditions such as hypoxia (a lack of oxygen) or anoxia (a lack of oxygen). Plant growth, development, and survival are severely hindered by these oxygen-deficient circumstances (Ashraf 2012).

**Fig. 2:**
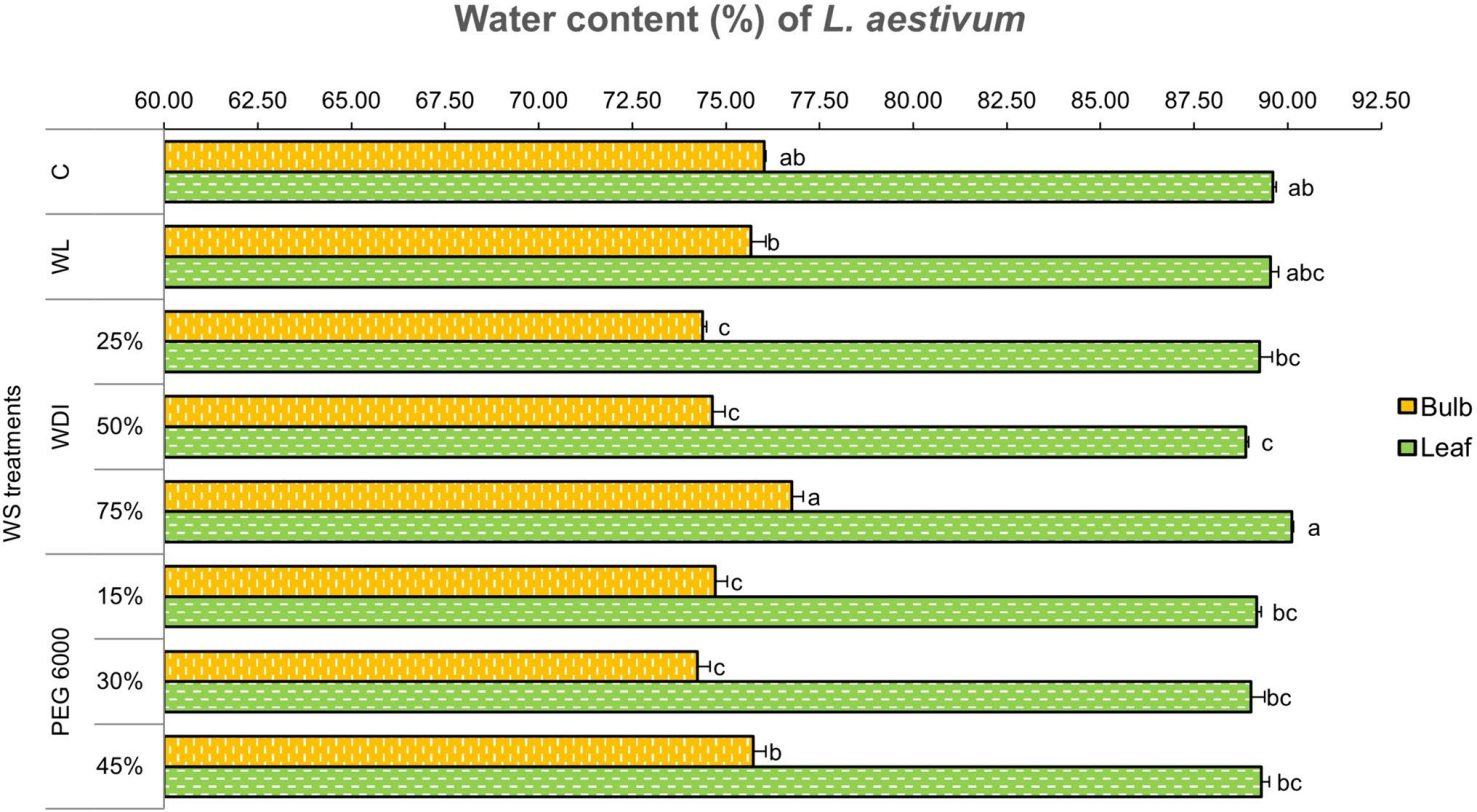
Effects of WS treatments on water content (%) in *L. aestivum* bulbs and leaves. Data are means ± standard error of three replicates (n = 3). Bars followed by different letters show significant difference according to Duncan test (*p* < 0.05). *Water content (%) = [Total fresh weight (g) − Total dry weight (g)]/Total fresh weight × 100]

It was observed that all WS treatments had lower values on the water content percentage in the bulbs and leaves compared to the control except 75% WDI group showing a slight increase by 1.4% in the bulbs and 0.5% in the leaves (Fig. 2). It was interesting that reducing IW by 75% did not reduce the water content; on the contrary, the bulb and leaves contained more water than the control, although there was no statistical difference. Even just 25% of normal IW did not reduce the water content of the plant, indicating that this plant was resistant to drought. Also, this plant was tolerant to WS, as evidenced by the fact that there was no sharp decrease in water content in any of the tested treatments. The lowest water content was obtained with 50% WDI treatment in the leaves, and 25% and 50% WDI, as well as 15% and 30% PEG 6000 in the bulbs, significantly compared to the control (Fig. 2).

Growth is one of the most water deficit-sensitive physiological processes because of the decline of turgor pressure. Cell expansion can occur only when the turgor pressure exceeds the cell wall yield threshold. Because of the low turgor pressure, WS substantially inhibits cell expansion and growth (Shao et al. 2008). Many studies showed that WS treatment had a negative correlation with growth parameters (De and Kar 1995; Hamed et al. 2022; Sun et al. 2010). Ates et al. (2021) reported that the treatment of salt stress, an abiotic stress factor, did not significantly change the shoot length, bulb size, and water content of *L. aestivum*. On the other hand, bulb widths and fresh weights were significantly increased with 45% PEG treatment in this study (Table 2). Similarly, leaf width and fresh weight were significantly raised with 75% DI and 15% PEG treatment, respectively. This could be explained by the fact that *L. aestivum* was more negatively impacted by salt stress in terms of growth and development.

There are various studies on the positive and negative effects of various WS on growth parameters. This response depends on the extent of WS or the plant species. For example, Seymen (2021) indicated that WL treatment reduced fresh weight, dry weight, and number of leaves, whereas it increased the leaf area of spinach at different harvest times. Barickman et al. (2019) found that cucumber plants subjected to the 10-day WL treatment decreased leaf area, leaf numbers, and fresh and dry mass. Ghodke et al. (2018) reported that WL stress adversely affected the plant growth like the plant height, leaf length and leaf area in onion (*Allium cepa* L.). Liu et al. (2021) exhibited that plant height, leaf length, and leaf area were not affected by WL stress in three cultivars of *Paeonia lactiflora* Pall. Manurung et al. (2019) evaluated the effect of drought stress on the growth parameters of *Ficus deltoidea* Jack using 4 different water field capacity (40%, 60%, 80% and 100%-C) levels. All stress levels reduced the plant height, leaf number, area, thickness, number of branches, and chlorophyll number. On the other hand, the 80% stress level increased the stem diameter and biomass while other stress levels reduced them in this plant. Jabeen et al. (2019) revealed that all the growth parameters (shoot fresh and dry weights and root fresh and dry weights) decreased with a 40% water deficit application in spinach. Weidner et al. (2009) recorded the highest dry matter content (22.7%) in the roots of *Vitis vinifera* L. under severe WS (35% soil moisture) when compared to the control (14.9%). Basha et al. (2015) determined a decline in root and shoot length with increasing PEG concentrations on the seedling development of tomato germplasm. Yosefi et al. (2022) determined that 7% PEG treatment had a detrimental impact on the strawberry plant's fresh and dry shoot and root weight. Bilir Ekbic et al. (2022) showed a negative effect on the shoot development of *Vitis labrusca* L. with increasing PEG doses.

### Analyses of galanthamine and lycorine contents by HPLC

The extraction yields and the results of the HPLC–DAD analysis of 16 different extracts obtained from *L. aestivum* bulbs and leaves were demonstrated in Table 3. The leaf extracts produced a greater yield rate than the bulb extracts when the extract yield percentages were compared. It could be a result of the chlorophyll and larger range of phenolic metabolites contained in the leaves.

**Table 3:** Effect of WS treatments on extraction yield (%) and alkaloid quantities (galanthamine and lycorine) (mg/g) in the bulb and leaf methanolic extracts of *L. aestivum* with HPLC–DAD analysis

WS treatments		Alkaloids in *L. aestivum* (mg/g dry extract)					
		Extraction yield (%)		Galanthamine		Lycorine	
		Bulb	Leaf	Bulb	Leaf	Bulb	Leaf
C	–	6.14	28.01	31.54 ± 0.36^c^	21.70 ± 0.03^e^	29.66 ± 0.21^e^	3.43 ± 0.00^ h^
WL	–	5.77	28.07	31.32 ± 0.11^c^	20.66 ± 0.25^f^	31.56 ± 0.09^d^	4.28 ± 0.05^f^
WDI	25%	6.20	28.16	28.76 ± 0.44^d^	20.97 ± 0.06^f^	27.54 ± 0.24^f^	3.67 ± 0.01^ g^
	50%	647	25.02	34.92 ± 0.06^a^	26.17 ± 0.02^a^	40.19 ± 0.01^a^	5.84 ± 0.00^b^
	75%	6.62	25.98	34.11 ± 1.30^ab^	13.68 ± 0.03^ g^	35.29 ± 0.69^c^	6.36 ± 0.01^a^
PEG 6000	15%	7.23	28.77	32.79 ± 0.09^bc^	24.77 ± 0.06^b^	37.33 ± 0.10^b^	4.96 ± 0.00^d^
	30%	7.28	29.49	31.99 ± 0.25^c^	24.12 ± 0.10^c^	29.48 ± 0.10^e^	4.45 ± 0.02^e^
	45%	7.32	26.92	32.16 ± 0.37^c^	23.43 ± 0.39^d^	31.25 ± 0.20^d^	5.06 ± 0.07^c^

Data are means ± standard error of three replicates (n = 3). Means with the different letters within columns show significant difference according to Duncan test (*p* < 0.05). Data expressed is as mg/g dry extract for alkaloids (galanthamine and lycorine)

**Fig. 3:**
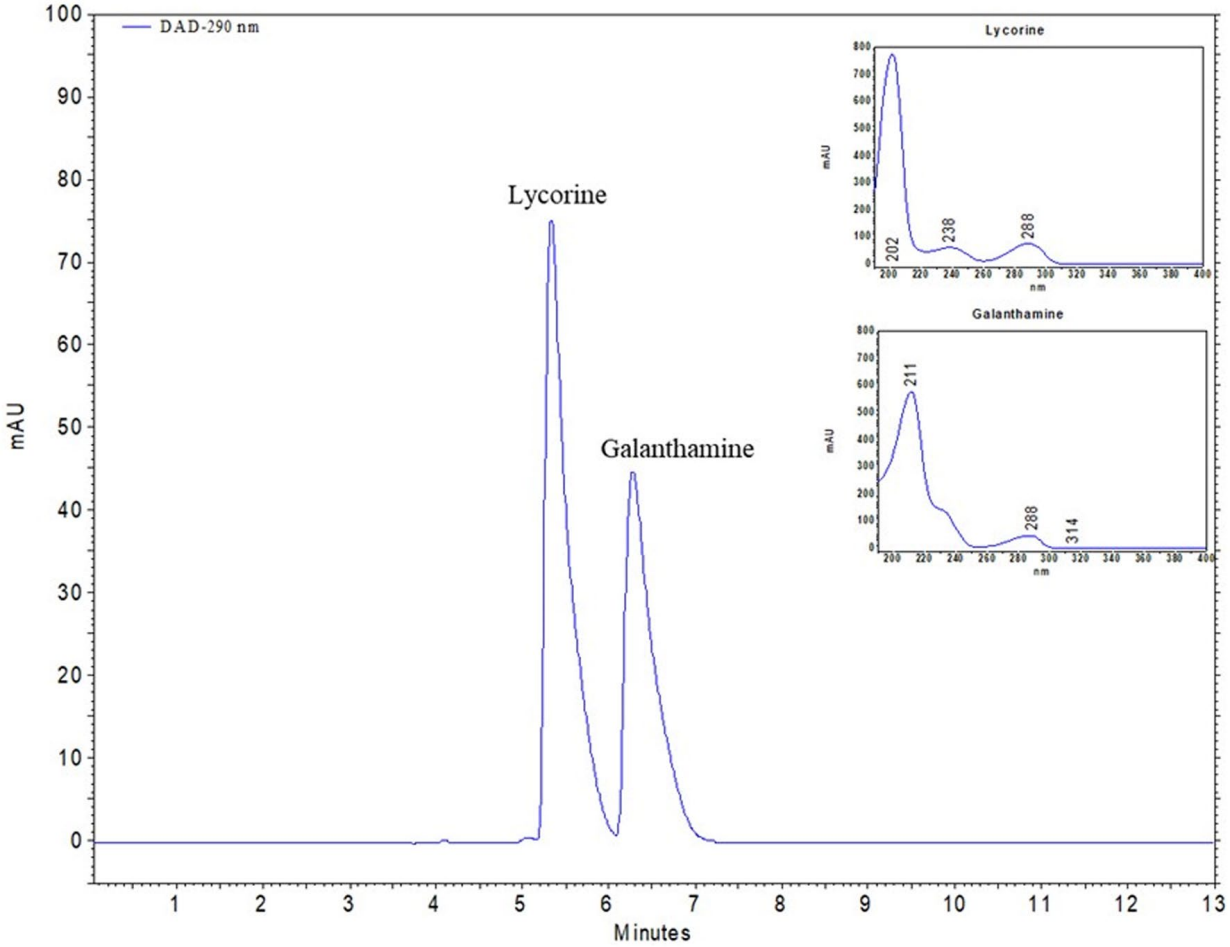
HPLC chromatogram of three replicates (n = 3) of alkaloid standards and their spectrums. Retention times: (1) lycorine-5.31 min, (2) galanthamine-6.29 min

**Fig. 4:**
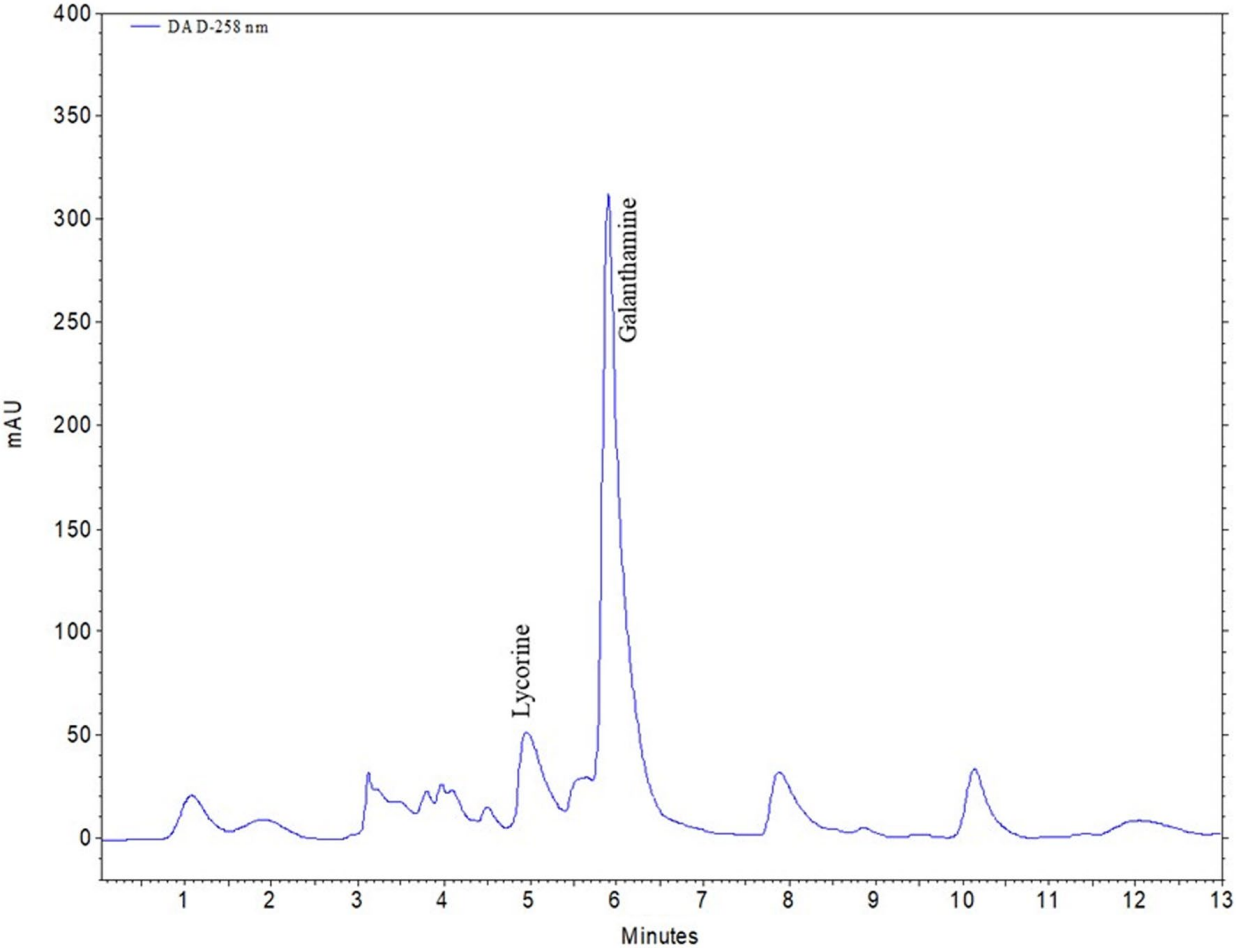
HPLC chromatogram of three replicates (n = 3) of the bulbs obtained from 50% WDI treatment

When the bulbs were evaluated among themselves, the PEG groups were on average about 18.5% higher than the control group. Figure 3 depicts the chromatogram of the utilized standards. Based on the results of an HPLC analysis of the bulb extracts, the treatment of 50% WDI resulted in the greatest concentrations of galanthamine and lycorine (Table 3; Fig. 4). 50% WDI increased galanthamine and lycorine levels by 10.72% and 35.50%, respectively, compared to the control (Table 3). Application of 75% WDI was also supported the galanthamine level, with an increase of 8.15% compared to the control. This was followed by 15% PEG treatment, which increased the amount of galantamine and lycorine in the bulbs by 3.96% and 25.86%, respectively, in comparison to the control.

**Fig. 5:**
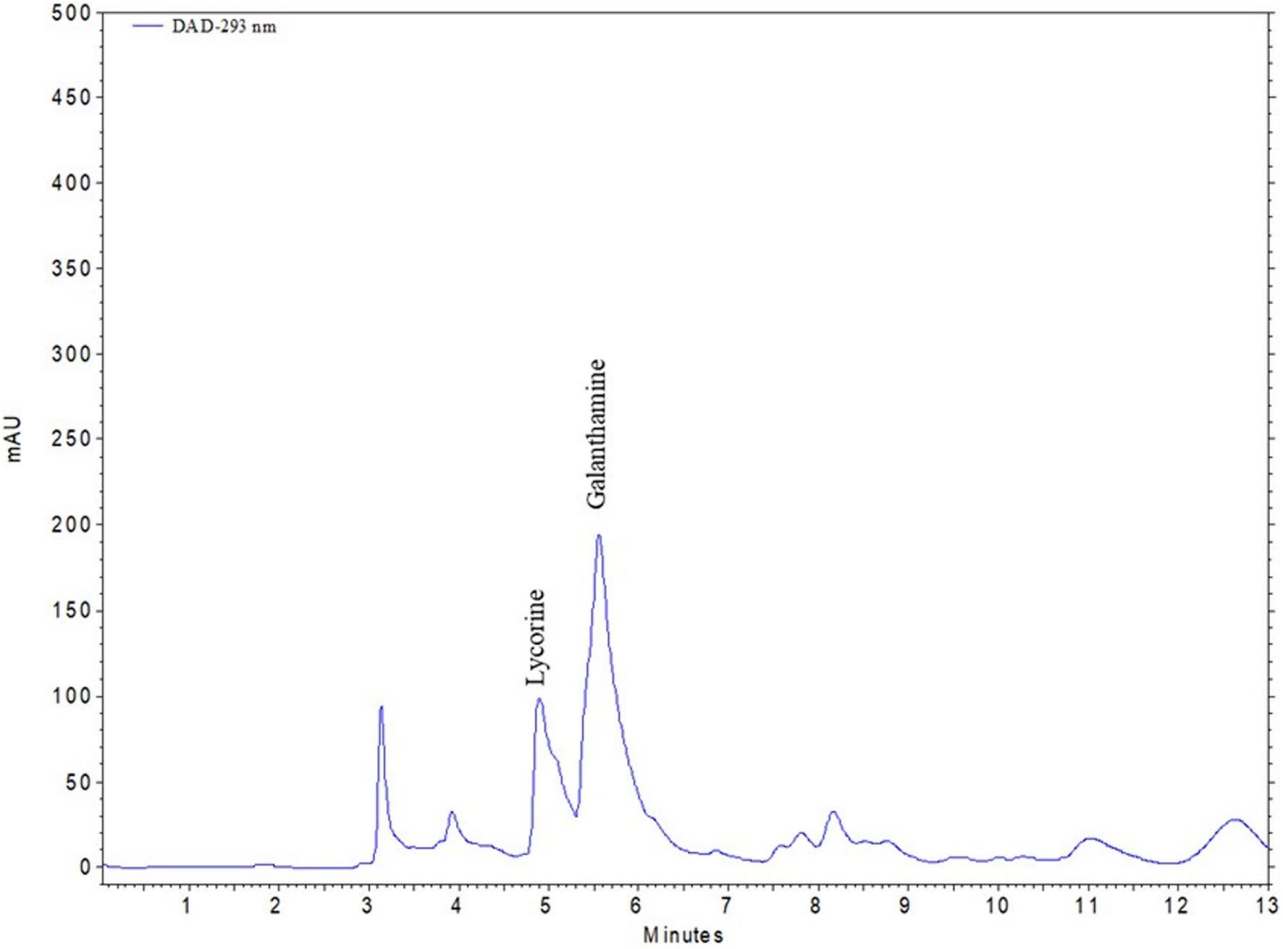
HPLC chromatogram of three replicates (n = 3) of the leaves obtained from 50% WDI treatment

In regard to HPLC analysis of the leaf extracts, it was observed that the amount of galanthamine was less than in the bulb extracts. When the leaves were evaluated within themselves, 50% WDI for the amount of galanthamine (20.60% rise) (Table 3; Fig. 5) and 75% WDI for the amount of lycorine (85.42% rise) came to the fore (Table 3). In addition, the mild WS (50% WDI) significantly enhanced the lycorine amounts (70.3% rise) in the leaves. There was an increase in galanthamine and lycorine amounts across the board for all PEG treatments (Table 3). Galanthamine levels in the leaves rose by 14.15, 11.5%, and 7.97%, respectively, following 15%, 30%, and 45% PEG treatments. Lycorine levels were elevated by 44.61%, 29.74%, and 47.52%, following the same treatments. While the WS brought on by WL was ineffective in raising the levels of bulb and leaf galanthamine, it was effective in raising the levels of bulb and leaf lycorine by 6.41% and 24.78%, respectively (Table 3).

In dry seasons as opposed to rainy seasons, alkaloid concentrations frequently tend to be higher. It is known that when plants are exposed to WS, the amounts of alkaloids in those plants rise (Gershenzon 1984). Alkaloid concentrations of *L. aestivum* were enhanced by certain WS applications herein study (Table 3), and several previous studies imposed the positive effect of drought stress on the alkaloid levels of *L. aestivum*. For example, Arslan et al. (2020) investigated the monthly variations of galanthamine and lycorine content in *L. aestivum*. It was found that the highest galanthamine and lycorine quantities were reported during the driest months, like July and August in Bolu, Turkey. Moreover, Demir et al. (2022) investigated the seasonal fluctuation in the alkaloid content of *L. aestivum* in the bulbs and leaves taken from 2 separate localities (Gölcük and Yeniçağa) in Bolu, Turkey, over the course of 8 months. In both locations, dry months like August showed a rise in the amounts of each alkaloid in the bulbs.

WS, in its broadest definition, includes both drought and salt stress. Salinity and drought both cause similar reactions in plants (Zhu 2002). For instance, the osmotic effect is the first step of salt stress and has many similarities to the effects of drought stress. In both situations, plants are unable to absorb enough water for appropriate growth and development, which causes the activation of signaling pathways connected to stress in plants (Uddin et al. 2016). Ates et al. (2021) investigated the influence of salt stress treatments on *L. aestivum*. They determined that the amounts of galanthamine and lycorine in the bulbs (28.5 mg/g and 69.5 mg/g dry extract, respectively) increased 4.13-fold and 1.39-fold in comparison to the control group when *L. aestivum* was treated for salt stress using 4 g/L CaCl_2_. They also found that the amount of galanthamine in the leaves was elevated with the treatment of 8 g/L NaCl and that the salinity stress they applied had no effect on the amount of lycorine in the leaves. When compared to the WS treatments in the current study (Table 3), 50% WDI gave a higher galanthamine amount in bulbs (34.92 mg/g dry extract) than the finding of Ates et al. (2021). However, salinity stress made a higher enhancement in alkaloid levels when compared to the control. Likewise, Ptak et al. (2019) determined that the salinity stress applied by using 100 mM NaCl in vitro increased galanthamine content 2.6 times compared to the control group.

Numerous investigations have shown that plants exposed to WS accumulated larger levels of secondary metabolites. The quantities of alkaloids such as trigonelline, pyrrolizidine alkaloids, quinolizidine alkaloids, steroid alkaloids, morphine alkaloids, indole alkaloids, nicotiana alkaloids, and benzylisoquinolines were found to rise in response to WS in many studies (Selmar and Kleinwächter 2013). Sahoo et al. (2012) found that total alkaloids of *Barleria prionitis, Boerhavia diffusa, Citrullus colocynthis*, and *Grewia tenax* significantly increased in the summer period when compared to the winter and rainy seasons. Guo et al. (2007) investigated different temperatures in short-term and long-term conditions on *Catharanthus rosesus* L. They found that high-temperature treatments increased the alkaloid content. Liu et al. (2017) investigated the effects of PEG-induced WS on the regulation of terpenoid indole alkaloid biosynthesis in *Catharanthus roseus*. As a result, they observed that vindoline and catharanthine contents were gradually enhanced and then reduced under 35% PEG 6000 stress, but vinblastine content gradually increased. Jaleel et al. (2008) found that the ajmalicine amount was enhanced in drought-stressed *C. roseus* when compared to the control plant.

### Non-enzymatic antioxidant activities

The half-maximal inhibitory concentration (IC_50_) was used to demonstrate the capacity to scavenge free radicals. In the experiment, quercetin was used as a reference. When the radical scavenging capacities of leaves and bulbs were evaluated, it was obvious that the bulbs had a larger capacity (Table 4). Stress treatments on leaves reduced their ability to scavenge radicals as compared to the control. With a 30.98% higher IC_50_ value than the control, 50% WDI in the bulbs had the greatest free radical scavenging activity (Table 4). Antioxidant capacity was also augmented with 75% WDI and 15% PEG by 15.60% and 13.74%, respectively, compared to control. Moreover, a small increase in antioxidant power (8.51%) was revealed when the WL stress was compared to the control (Table 4).

**Table 4:** Effect of WS treatments on DPPH radical scavenging activity, and TPC and TFC values in *L. aestivum*

WS treatments		*L. aestivum* extracts					
		IC_50_ DPPH inhibition (mg/ml)		Total phenol (mg GAE/g extract)		Total flavonoid (mg QE/g extract)	
		Bulb	Leaf	Bulb	Leaf	Bulb	Leaf
C	–	7.78 ± 0.13^f^	10.60 ± 0.11^b^	25.78 ± 0.87^e^	10.48 ± 0.33^abc^	2.59 ± 0.05^bc^	63.58 ± 0.27^d^
WL	–	7.17 ± 0.10^d^	13.08 ± 0.23^e^	28.90 ± 0.41^ cd^	6.96 ± 0.19^d^	2.62 ± 0.08^bc^	64.64 ± 0.27^c^
WDI	25%	7.49 ± 0.00^e^	11.40 ± 0.12^c^	30.28 ± 0.37^bc^	9.58 ± 0.58^c^	2.62 ± 0.08^bc^	61.77 ± 0.07^e^
	50%	5.94 ± 0.09^b^	11.24 ± 0.19^c^	34.30 ± 0.15^a^	9.85 ± 0.30^bc^	3.74 ± 0.02^a^	75.17 ± 0.35^a^
	75%	6.73 ± 0.03^c^	11.30 ± 0.03^c^	31.73 ± 0.92^b^	11.20 ± 0.31^ab^	2.74 ± 0.05^b^	62.59 ± 0.35^e^
PEG 6000	15%	6.84 ± 0.02^c^	14.61 ± 0.37^f^	27.08 ± 0.81^de^	11.71 ± 0.44^a^	2.21 ± 0.12^d^	69.41 ± 0.35^b^
	30%	7.88 ± 0.14^f^	12.29 ± 0.01^d^	30.11 ± 1.11^bc^	10.28 ± 0.37^bc^	2.44 ± 0.05^ cd^	60.02 ± 0.50^f^
	45%	7.73 ± 0.08^f^	12.68 ± 0.12^de^	26.21 ± 0.06^e^	11.19 ± 0.54^ab^	2.21 ± 0.12^d^	62.44 ± 0.11^e^
Quercetin	–	0.04 ± 0.00^a^	0.03 ± 0.00^a^				

Data are means ± standard error of three replicates (n = 3). Means with the different letters within columns show significant difference according to Duncan test (*p* < 0.05)

Due to the hydroxyl groups on their structures, phenols and flavonoids are strong free radical scavengers. The total phenol and flavonoid contents (TPC and TFC) of all methanol extracts are indicated in Table 4. The calibration curve for gallic acid (R^2^ = 0.999) was utilized to calculate the TPC of the *L. aestivum* bulb and leaf extracts. All of the WS treatments significantly increased the TPC of the bulbs. In particular, WDI treatments were more effective in the increment of the bulb TPC. The best enhancement was obtained with 50% WDI treatment, showing a 33.05% rise compared to the control. Furthermore, 75% WDI treatment increased the TPC of the bulbs by 23.08%. In contrast to TPC in the bulb, TPC in the leaf showed the best increase with 15% PEG treatment (11.74% rise) when compared to the control. Although WL stress caused the TPC value of the bulb to slightly extend by 12.10%, the TPC value of the leaf decreased by 50.57% (Table 4).

The quercetin calibration curve (R^2^ = 0.999) was utilized to identify the TFC of *L. aestivum* extracts. Similar to the TPC value in the bulbs, the 50% WDI treatment produced the greatest increase in bulb TFC value (44.40% rise), followed by the 75% WDI treatment (5.79% rise). The TFC values of the bulbs were actually decreased by the WS that the PEG induced, rather than being successful in raising it. Also, there was no significant difference between the WL group and the control. Leaf TFC value enhancement was supported by 50% DI (18.23% rise), followed by 15% PEG (9.17% rise). WL stress caused a slight increase (1.67%) in the TFC value in the leaves (Table 4).

IC_50_ values of all WS treatments in the bulbs indicated a strong negative correlation with TPC and TFC values (r =  − 0.79 and − 0.74, respectively, *P* < 0.05), and it was possible to conclude that the increase in TPC and TFC generated by the WS treatments resulted in an increase in antioxidant capacity.

In the study of Ates et al. (2021), all salt stress applications did not increase the antioxidant power in the bulbs, but some concentrations enhanced the antioxidant capacity in the leaves. On the other hand, in the present study, WS applications significantly elevated the antioxidant capacity in the bulbs, but the antioxidant force in the leaves was reduced with all WS activations (Table 4). In parallel with the results of the current study, Ates et al. (2021) reported that some salt stress applications in *L. aestivum* increased the TPC and TFC values of both bulbs and leaves. According to Demir et al. (2022), the dry months with the maximum temperatures (July and August) in Bolu localities (Gölcük and Yeniçağa) also caused the greater content of total phenol and antioxidant capacity in the bulbs, which was parallel to the results of the measured alkaloid concentrations.

WS has often been demonstrated to boost antioxidant potency and phenolic content in a wide range of plant species but has also been reported to reduce them in some cases. For example, Weidner et al. (2009) indicated the highest TPC of grapevines in the roots with severe WS conditions (35% soil moisture) compared to the control. Ferulic acid, p-coumaric acid, and caffeic acid all showed the highest increases. In contrast, they found a decline in the DPPH radical scavenging activity of grapevine plants with severe WS conditions (35% soil moisture). Bettaieb Rebey et al. (2012) showed that moderate and severe WS conditions increased the TPC and IC_50_ values of cumin. Gharibi et al. (2016) determined that TFC values and DPPH scavenging activity of *A. filipendulina*, *A. millefolium* and *A. nobilis* were enhanced by under severe drought conditions (25% field capacity). Popović et al. (2016) applied WS (100 and 200 mOsm PEG 6000) on three poplar (*Populus deltoides* L.) genotypes (M1, B229, and PE19/66) for six days. They found a significant increase in antioxidant activity in the B229 leaf under 100 mOsm PEG 6000 stress treatment. On the other hand, TPC of all poplar genotypes was reduced under WS applications. Sarker and Oba (2018) found that severe WS conditions increased the DPPH scavenging activity and total phenolic-flavonoid content of *Amaranthus tricolor* L. The highest content of salicylic acid, vanillic acid, gallic acid, chlorogenic acid, and p-hydroxybenzoic acid with moderate and severe WS conditions was also reported. Ghodke et al. (2018) indicated a drastic reduction in phenol-flavonoid content and antioxidant activity at the end of the WL stress condition in onion (*A. cepa*).

### Enzymatic antioxidant activities

The superoxide dismutase (SOD) metalloenzyme is found in nearly every aerobic species and serves as an essential antioxidant enzyme in all subcellular sections susceptible to ROS-assisted oxidative damage. Abiotic stresses induce the formation of ROS; in this case, SOD functions as an initial defense system, raising the plant's resistance to the harmful effects of ROS by catalyzing the O_2_^•−^ into H_2_O_2_ and O_2_ (Hussain et al. 2019). The alterations in the SOD and CAT activities of *L. aestivum* bulbs and leaves under various WS stress were demonstrated in Table 5. WS applications produced by WL and 30% PEG resulted in the highest SOD activity in the bulbs and leaves, with 8.33% and 3.28% increases, respectively. WS generated by water deficiency (WDI treatments) was not effective to increase the SOD activity of the bulbs and leaves (Table 5).

**Table 5:** Effect of WS treatments on SOD and CAT activity in *L. aestivum*

WS treatments		*L. aestivum*			
		SOD activity (U/mg protein)		CAT activity (mmol/min/mg protein)	
		Bulb	Leaf	Bulb	Leaf
C	–	0.096 ± 0.000^bc^	0.061 ± 0.000^ab^	6.213 ± 0.732^d^	29.111 ± 0.945^ cd^
WL	–	0.104 ± 0.000^a^	0.059 ± 0.000^bc^	8.886 ± 1.451^ cd^	31.456 ± 0.802^bc^
WDI	25%	0.090 ± 0.001^d^	0.054 ± 0.000^d^	13.523 ± 1.041^ab^	30.285 ± 1.071^ cd^
	50%	0.085 ± 0.001^e^	0.050 ± 0.000^e^	9.105 ± 0.326^ cd^	22.152 ± 0.694^f^
	75%	0.081 ± 0.000^f^	0.057 ± 0.000^c^	15.512 ± 1.242^a^	36.284 ± 1.833^a^
PEG 6000	15%	0.095 ± 0.001^bc^	0.052 ± 0.000^de^	15.583 ± 1.113^a^	26.856 ± 0.725^de^
	30%	0.097 ± 0.001^b^	0.063 ± 0.001^a^	10.576 ± 2.001^bc^	25.392 ± 1.044^ef^
	45%	0.094 ± 0.000^c^	0.057 ± 0.001^c^	10.272 ± 0.545^bcd^	34.045 ± 1.442^ab^

Data are means ± standard error of three replicates (n = 3). Means with the different letters within columns show significant difference according to Duncan test (*p* < 0.05)

Catalase (CAT) enzymes contain tetrameric heme, giving them the tendency to react with H_2_O_2_ and convert it into H_2_O and O_2_. CATs are important enzymes for the detoxification of ROS under stress conditions because CAT has the highest runover rates of all enzymes (Hussain et al. 2019). Increased CAT activity was determined in the bulbs with all WS treatments. Notable increases in CAT activities (2.50-fold) in the bulbs were observed with 15% PEG and 75% WDI treatments in comparison to the control group (Table 5). Similarly, the highest CAT activity in the leaves was determined with the 75% WDI treatment (24.64% elevation) when compared to the control. In addition, applying 45% PEG effectively increased the CAT activity in leaves by 16.95% in comparison to the control (Table 5).

Some previous studies showed the enhanced activity of antioxidant enzymes in *L. aestivum* in response to salt stress (Ptak et al. 2019; Ates et al. 2021). Ptak et al. (2019) found that only 50 and 150 mM NaCl treatments enhanced SOD activity, but all tested doses of NaCl (50, 100, 150, and 200 mM) increased CAT activity in in vitro*-*grown *L. aestivum*. Similarly, salt stress treatments in pot culture at various concentrations of NaCl and CaCl_2_ resulted in increased antioxidant enzyme activity in the bulbs and leaves of *L. aestivum,* according to Ates et al. (2021).

Various abiotic stresses increase the antioxidant enzyme activities like SOD and CAT in numerous plants. Stress-induced variation of antioxidants relies on the severity and duration of the treatment and the species and age of the plant (Pan et al. 2006). Yosefi et al. (2022) observed that PEG-induced WS led to higher activity in the SOD and POD (peroxidase) enzymes at 7% PEG treatment on *Fragaria* × *ananassa*. Batool et al. (2022) found that 15% PEG 6000 treatment increased the SOD and CAT activity of rapeseed cultivars. However, drought-tolerant cultivars of rapeseeds increased more than sensitive cultivars. Jaleel et al. (2008) determined that SOD activity increased in all WS of 10-, 15-, and 20-days interval drought on *C. roseus* roots. Pan et al. (2006) indicated that PEG 6000-induced WS decreased the SOD and CAT antioxidant enzyme activities except for POD activity in *Glycyrrhiza uralensis* L. Yuan et al. (2016) generated different levels of WS (control-75 to 80% of field water capacity, mild-55% to 60%, moderate-45% to 50%, and severe WS- 35 to 40%) on tomato plants. They found that antioxidant enzyme activities were increased with the increasing degree of WS.

Despite a minor decrease in SOD activity in the leaves, there was an increase in SOD activity in the bulbs, as well as in both enzyme activities (CAT and SOD) in the bulbs and leaves under WL stress in the current study (Table 5). Various studies have also indicated that WL type stress can alter the activity of antioxidant enzymes in different plant species. As an example, Yan et al. (1996) reported a decreased SOD activity with WL stress on corn leaves. Ahmed et al. (2002) determined that SOD, CAT, APX, and GR were reduced during prolonged WL treatment on mungbean. Kumutha et al. (2009) determined a rise in the antioxidant enzymes such as SOD, APX, GR, and CAT increased with WL stress in pigeon pea plants. Wang et al. (2019) indicated that the antioxidant enzyme activity (CAT, SOD, and POD) of *Triarrhena sacchariflora* Nakai was increased with WL treatments. Liu et al. (2021) showed an increment in the activity of antioxidant enzymes (SOD, POD, and CAT) in all varieties of *P. lactiflora* under WL stress.

For plants, water deficit situations lead to the overproduction of ROS, which results in growth inhibition, in photosynthetic functions, lipid peroxidation, and programmed cell death. Nevertheless, plants have developed several acclimation strategies to adapt to WS, which include osmotic adjustment and antioxidant defense systems, which increase their ability to grow and thrive in drought circumstances (Sun et al. 2020). In the present study, the increase in antioxidant enzyme activities with WS treatments was a reaction to oxidative stress, especially PEG-induced WS caused higher SOD and CAT activities in the bulbs comparing to water deficiency stress. It is interesting that 50% WDI showed lower SOD activity in the bulbs and leaves, and CAT activity was moderately increased in the bulbs and reduced in the leaves compared to the control. In connection with this result, moderate water deficit level (50% WDI) produced the highest galanthamine content in the bulbs and leaves. These findings showed that *L. aestivum* increased galanthamine levels without being exposed to too much stress.

Galanthamine levels in response to WDI-induced WS showed a negative correlation with SOD or CAT activity in the bulbs (r =  − 0.84 and r =  − 0.33, respectively, *P* < 0.05) and in the leaves (r =  − 0.98 for both, *P* < 0.05). It may be inferred that increased galantamine levels resulted from decreased SOD and CAT enzyme activities.

Elevated SOD and CAT activities of the bulbs in WL condition showed that *L. aestivum* experienced additional stress in a submerged state. However, the additional stress reduced growth performance and might have been too much to promote alkaloid bioaccumulation and non-enzymatic antioxidant properties. A slight increase in water content with 75% WDI-induced WS in both bulbs and leaves may be associated with the most increased CAT activities at this stress level. Although PEG-induced WS had more positive effects on growth parameters, this type of WS imposed more stress, as obviously seen from increased CAT levels. WS applications generated by 50% WDI enhanced the bulb width, and the most enhanced galanthamine quantity, antioxidant capacity, and total phenol-flavonoid content were obtained with this stress type with moderately elevated CAT activity (46.55%) when compared to control. In regard to all findings, mild water deficiency (50% WDI) had advantages over the other treatments.

## Conclusion

This study revealed for the first time the impacts of eight different WS treatments on the accumulation of alkaloids, antioxidant capacity, and growth parameters in *L. aestivum*. Treatment of 45% PEG significantly increased the bulb widths and fresh weights. Treatment of 75% WDI notably elevated the leaf widths, whereas 15% PEG treatment increased the leaf fresh weights. Among the WS treatments, galanthamine and lycorine levels in the bulbs were improved by 50% WDI treatment. As well as antioxidant capacity, and total phenol-flavonoid content in the bulbs were superior with 50% WDI. *L. aestivum* is a drought- and stress-tolerant plant as a result of the enhanced activity of antioxidant defense enzymes. Moderately elevated CAT activity was provided by moderate water deficiency (50% WDI) associated with the highest alkaloid quantities and antioxidant power. The elevated SOD and CAT activities of the bulbs in the WL condition indicated that *L. aestivum* was subjected to extra stress in a submerged state. When all outcomes were considered together, this plant can be cultivated under moderate water deficiency to increase the medicinal quality of the bulbs, especially in terms of galanthamine and antioxidant capacity. *L. aestivum* can be easily planted in drought-stricken areas to increase the production of galanthamine. Future studies should focus on the mechanism of various WS applications on alkaloid accumulation in *L. aestivum*.

## References

[CR1] Ahmed S, Nawata E, Hosokawa M, Domae Y, Sakuratani T (2002). Alterations in photosynthesis and some antioxidant enzymatic activities of mungbean subjected to waterlogging. Plant Sci.

[CR2] Ahsan N, Lee DG, Lee SH, Kang KY, Bahk JD, Choi MS, Lee IJ, Renaut J, Lee BH (2007). A comparative proteomic analysis of tomato leaves in response to waterlogging stress. Physiol Plant.

[CR3] Aroca R, Porcel R, Ruiz-Lozano JM (2012). Regulation of root water uptake under abiotic stress conditions. J Exp Bot.

[CR4] Arslan M, Yildirim AB, Ozkan E, Oktelik FB, Turker AU (2020). Monthly variation of pharmaceutically valuable alkaloids, galanthamine and lycorine, in summer snowflake (*Leucojum aestivum* L.). Fresenius Environ Bull.

[CR5] Ashraf MA (2012). Waterlogging stress in plants: A review. Afr J Agric Res.

[CR6] Ates MT, Yildirim AB, Turker AU (2021). Enhancement of alkaloid content (galanthamine and lycorine) and antioxidant activities (enzymatic and non-enzymatic) under salt stress in summer snowflake (*Leucojum aestivum* L.). S Afr J Bot.

[CR7] Barickman TC, Simpson CR, Sams CE (2019). Waterlogging causes early modification in the physiological performance, carotenoids, chlorophylls, proline, and soluble sugars of cucumber plants. Plants.

[CR8] Basha PO, Sudarsanam G, Reddy MMS, Sankar S (2015). Effect of PEG-induced water stress on germination and seedling development of tomato germplasm. Inter J Recent Sci Res.

[CR9] Batool M, El-Badri AM, Wang Z, Mohamed IAA, Yang H, Ai X, Salah A, Hassan MU, Sami R, Kuai J, Wang B, Zhou G (2022). Rapeseed morpho-physio-biochemical responses to drought stress induced by PEG-6000. Agronomy.

[CR10] Bettaieb Rebey I, Jabri-Karoui I, Hamrouni-Sellami I, Bourgou S, Limam F, Marzouk B (2012). Effect of drought on the biochemical composition and antioxidant activities of cumin (*Cuminum cyminum* L.) seeds. Ind Crops Prod.

[CR11] Bilir Ekbic H, Gecene İ, Ekbic E (2022). Determination of the tolerance of fox grapes (*Vitis**labrusca* L.) to drought stress by PEG application in vitro. Erwerbs-Obstbau.

[CR12] Blois MS (1958). Antioxidant determinations by the use of a stable free radical. Nature.

[CR13] Buxton DR, Fales SL (1994) Plant Environment and Quality. In: Fahey Jr GC (ed) Forage Quality, Evaluation, and Utilization. America, pp 155–199

[CR14] De R, Kar RK (1995). Seed germination and seedling growth of mung bean (*Vigna radiata*) under water stress induced by PEG-6000. Seed Science and Technology.

[CR15] Deeba F, Pandey AK, Ranjan S, Mishra A, Singh R, Sharma YK, Shirke PA, Pandey V (2012). Physiological and proteomic responses of cotton (*Gossypium herbaceum* L.) to drought stress. Plant Physiol Biochem.

[CR16] Demir SC, Yildirim AB, Turker AU, Eker I (2022). Seasonal variation in alkaloid content, phenolic constituent, and biological activities of some *Leucojum aestivum* L. populations in Turkey. S Afr J Bot.

[CR17] Diop MF, Hehn A, Ptak A, Chrétie F, Doerper S, Gontier E, Bourgaud F, Henry M, Chapleur Y, Laurain-Mattar D (2007). Hairy root and tissue cultures of *Leucojum aestivum* L. relationships to galanthamine content. Phytochem Rev.

[CR18] Farooq M, Wahid A, Kobayashi N, Fujita D, Basra SMA (2009). Plant drought stress: effects, mechanisms, and management. Agron Sustain Dev.

[CR19] Gershenzon J, Timmermann BN, Steelink C, Loewus FA (1984). Changes in the levels of plant secondary metabolites under water and nutrient stress. Phytochemical adaptations to stress.

[CR20] Gharibi S, Tabatabaei BES, Saeidi G, Goli SAH (2016). Effect of drought stress on total phenolic, lipid peroxidation, and antioxidant activity of *Achillea* species. Appl Biochem Biotechnol.

[CR21] Ghodke PH, Shirsat DV, Thangasamy A, Mahajan V, Salunkhe VN, Khade Y, Singh M (2018). Effect of water logging stress at specific growth stages in onion crop. Int J Curr Microbiol Appl Sci.

[CR22] Guo X, Yang L, Yu J, Tang Z, Zu Y (2007). Alkaloid variations in *Catharanthus roseus* seedlings treated by different temperatures in short term and long term. J for Res.

[CR23] Gutiérrez-Miceli FA, Santiago-Borraz J, Montes Molina JA, Nafate CC, Abud-Archila M, Oliva Llaven MA, Rincón-Rosale R, Dendooven L (2007). Vermicompost as a soil supplement to improve growth, yield and fruit quality of tomato (*Lycopersicum esculentum*). Bioresour Technol.

[CR24] Hamed SB, Lefi E, Chaieb M (2022). Growth phenology of pistachio seedlings under water stress and rehydration conditions. J Plant Biol Crop Res.

[CR25] Hussain S, Rao MJ, Anjum MA, Ejaz S, Zakir I, Ali MA, Ahmad N, Ahmad S, Hasanuzzaman M, Hakeem K, Nahar K, Alharby H (2019). Oxidative stress and antioxidant defense in plants under drought conditions. Plant abiotic stress tolerance.

[CR26] Ivanov I, Berkov S, Pavlo A, Georgiev V (2019). In sito galanthamine extraction during the cultivation of *Leucojum aestivum* L. shoot culture in two-phase bubble column cultivation system. Eng Life Sci.

[CR27] Jabeen M, Akram NA, Ashraf M, Aziz A (2019). Assessment of biochemical changes in spinach (*Spinacea**oleracea* L.) subjected to varying water regimes. Sains Malays.

[CR28] Jaleel CA, Sankar B, Murali PV, Gomathinayagam M, Lakshmanan GMA, Panneerselvam R (2008). Water deficit stress effects on reactive oxygen metabolism in *Catharanthus roseus*; impacts on ajmalicine accumulation. Colloids Surf B.

[CR29] Jaleel CA, Manivannan P, Wahid A, Farooq M, Al-Juburi HJ, Somasundaram R, Panneerselvam R (2009). Drought stress in plants: a review on morphological characteristics and pigments composition. Int J Agric Biol.

[CR30] Jiang M (2002). Water stress-induced abscisic acid accumulation triggers the increased generation of reactive oxygen species and up-regulates the activities of antioxidant enzymes in maize leaves. J Exp Bot.

[CR31] Jin YH, Min JS, Jeon S, Lee J, Kim S, Park T, Park D, Jang MS, Park CM, Song JH, Kim HR, Kwon S (2021). Lycorine, a non-nucleoside RNA dependent RNA polymerase inhibitor, as potential treatment for emerging coronavirus infections. Phytomedicine.

[CR32] Kumutha D, Ezhilmathi K, Sairam RK, Srivastava GC, Deshmukh PS, Meena RC (2009). Waterlogging induced oxidative stress and antioxidant activity in pigeon pea genotypes. Biol Plant.

[CR33] Lartillot S, Kedziora P, Athias A (1988). Purification and characterization of a new fungal catalase. Prep Biochem.

[CR34] Liu Y, Meng Q, Duan X, Zhang Z, Li D (2017). Effects of PEG-induced drought stress on regulation of indole alkaloid biosynthesis in *Catharanthus roseus*. J Plant Interact.

[CR35] Liu M, Zhang Q, Xu J, Bao M, Zhang D, Xie A, Sun X (2021). Effects of waterlogging stress on the physiological characteristics and secondary metabolites of Herbaceous Peony (*Paeonia lactiflora* Pall.). Am J Plant Sci.

[CR36] Lowry OH (1951). Protein measurement with the folin phenol reagent. J Biol Chem.

[CR37] Mahajan S, Tuteja N (2005). Cold, salinity and drought stresses: an overview. Arch Biochem Biophys.

[CR38] Manurung H, Kustiawan W, Kusuma IW, Marjenah M, Nugroho RA (2019). Growth, phytochemical profile, and antioxidant activity of cultivated tabat barito (*Ficus deltoidea* Jack) under drought stress. Int J Biosci.

[CR39] Michel BE, Kaufmann MR (1973). The Osmotic Potential of Polyethylene Glycol 6000. Plant Physiol.

[CR40] Mill RR (1984) Flora of Turkey and the East Aegean Islands. In: Davis PH (ed) University Press, Edinburgh, pp 364–365

[CR41] Muscolo A, Sidari M, Anastasi U, Santonoceto C, Maggio A (2014). Effect of PEG-induced drought stress on seed germination of four lentil genotypes. J Plant Interact.

[CR42] Pan Y, Wu LJ, Yu ZL (2006). Effect of salt and drought stress on antioxidant enzymes activities and SOD isoenzymes of liquorice (*Glycyrrhiza uralensis* Fisch). Plant Growth Regul.

[CR43] Pavlov A, Berkov S, Courot E, Gocheva T, Tuneva D, Pandova B, Georgiev M, Georgiev V, Yanev S, Burrus M, Ilieva M (2007). Galanthamine production by *Leucojum aestivum* in vitro systems. Process Biochem.

[CR44] Popović BM, Štajner D, Ždero-Pavlović R, Tumbas-Šaponjac V, Čanadanović-Brunet J, Orlović S (2016). Water stress induces changes in polyphenol profile and antioxidant capacity in poplar plants (*Populus* spp.). Plant Physiol Biochem.

[CR45] Ptak A, Simlat M, Morańska E, Skrzypek E, Warchoł M, Tarakemeh A, Laurain-Mattar D (2019). Exogenous melatonin stimulated Amaryllidaceae alkaloid biosynthesis in in vitro cultures of *Leucojum aestivum* L. Ind Crops Prod.

[CR46] Saglam A, Saruhan N, Terzi R, Kadioglu A (2011). The relations between antioxidant enzymes and chlorophyll fluorescence parameters in common bean cultivars differing in sensitivity to drought stress. Russ J Plant Physiol.

[CR47] Sahoo KP, Kasera PK, Mohammed S (2012). Secondary metabolites produced during different seasons in some arid medicinal plants. Asian J Plant Sci Res.

[CR48] Saliba S, Ptak A, Laurain-Mattar D (2015). 4′-O-Methylnorbelladine feeding enhances galanthamine and lycorine production by *Leucojum aestivum* L. shoot cultures. Eng Life Sci.

[CR49] Sarker U, Oba S (2018). Drought stress enhances nutritional and bioactive compounds, phenolic acids and antioxidant capacity of *Amaranthus* leafy vegetable. BMC Plant Biol.

[CR50] Selmar D, Kleinwächter M (2013). Stress enhances the synthesis of secondary plant products: the impact of stress-related over-reduction on the accumulation of natural products. Plant Cell Physiol.

[CR51] Seymen M (2021). How does the flooding stress occurring in different harvest times affect the morpho-physiological and biochemical characteristics of spinach?. Sci Hortic.

[CR52] Shao HB, Chu LY, Jaleel CA, Zhao CX (2008). Water-deficit stress-induced anatomical changes in higher plants. C R Biol.

[CR53] Sun YY, Sun YJ, Wang MT, Li XY, Guo X, Hu R, Ma J (2010). Effects of seed priming on germination and seedling growth under water stress in rice. Acta Agron Sin.

[CR54] Sun Y, Wang C, Chen HY, Ruan H (2020). Response of plants to water stress: a meta-analysis. Front Plant Sci.

[CR55] Tewari S, Mishra A, Mohammad AA, Vijay PS, Durgesh KT, Pravej A, Mohammed NA (2018). Flooding stress in plants and approaches to overcome, in: Plant metabolites and regulation under environmental stress. Parvaiz A.

[CR56] Turker AU, Yıldırım AB, Tas I, Ozkan E, Turker H (2021). Evaluation of some traditional medicinal plants: phytochemical profile, antibacterial and antioxidant potentials. Rom Biotechnol Lett.

[CR57] Uddin MN, Hossain MA, Burritt DJ (2016). Salinity and drought stress: similarities and differences in oxidative responses and cellular redox regulation. Water Stress Crop Plants: Sustain Approach.

[CR58] Van den Berg L, Zeng YJ (2006). Response of South African indigenous grass species to drought stress induced by polyethylene glycol (PEG) 6000. S Afr J Bot.

[CR59] Van Rossum MWPC, Alberda M, van der Plas LHW (1997). Role of oxidative damage in tulip bulb scale micropropagation. Plant Sci.

[CR60] Wang J, Sun H, Sheng J, Jin S, Zhou F, Hu Z, Diao Y (2019). Transcriptome, physiological and biochemical analysis of *Triarrhena sacchariflora* in response to flooding stress. BMC Genet.

[CR61] Weidner S, Karolak M, Karamac M, Kosinska A, Amarowicz R (2009). Phenolic compounds and properties of antioxidants in grapevine roots [*Vitis vinifera* L.] under drought stress followed by recovery. Acta Soc Bot Pol.

[CR62] Yadav B, Jogawat A, Rahman MS, Narayan OP (2021). Secondary metabolites in the drought stress tolerance of crop plants: A review. Gene Rep.

[CR63] Yan B, Dai Q, Liu X, Huang S, Wang Z (1996). Flooding-induced membrane damage, lipid oxidation and activated oxygen generation in corn leaves. Plant Soil.

[CR64] Yang L, Wen KS, Ruan X, Zhao YX, Wei F, Wang Q (2018). Response of plant secondary metabolites to environmental factors. Mol.

[CR65] Yang X, Lu M, Wang Y, Wang Y, Liu Z, Chen S (2021). Response mechanism of plants to drought stress. Horticulturae.

[CR66] Yosefi A, Mozafari AA, Javadi T (2022). In vitro assessment of strawberry (*Fragaria× ananassa* Duch.) plant responses to water shortage stress under nano-iron application. In Vitro Cell Dev Biol Plant.

[CR67] Yuan XK, Yang ZQ, Li YX, Liu Q, Ha W (2016). Effects of different levels of water stress on leaf photosynthetic characteristics and antioxidant enzyme activities of greenhouse tomato. Photosynthetica.

[CR68] Zhang Y, Liu G, Dong H, Li C (2021). Waterlogging stress in cotton: damage, adaptability, alleviation strategies, and mechanisms. Crop J.

[CR69] Zhu JK (2002). Salt and drought stress signal transduction in plants. Annu Rev Plant Biol.

